# RGS5: a novel role as a hypoxia-responsive protein that suppresses chemokinetic and chemotactic migration in brain pericytes

**DOI:** 10.1242/bio.059371

**Published:** 2022-10-17

**Authors:** Andreas Enström, Robert Carlsson, Ilknur Özen, Gesine Paul

**Affiliations:** ^1^ Translational Neurology Group, Department of Clinical Science, Lund University, Lund 221 84, Sweden; ^2^ Department of Neurology, Scania University Hospital, Lund 221 85, Sweden; ^3^ Wallenberg Centre for Molecular Medicine, Lund University, Lund 221 84, Sweden

**Keywords:** Hypoxia, Migration, PDGFBB, Pericytes, RGS5, S1P

## Abstract

Adaptive biological mechanisms to hypoxia are crucial to maintain oxygen homeostasis, especially in the brain. Pericytes, cells uniquely positioned at the blood-brain interface, respond fast to hypoxia by expressing regulator of G-protein signalling 5 (RGS5), a negative regulator of G-protein-coupled receptors. RGS5 expression in pericytes is observed in pathological hypoxic environments (e.g. tumours and ischaemic stroke) and associated with perivascular depletion of pericytes and vessel leakage. However, the regulation of RGS5 expression and its functional role in pericytes are not known. We demonstrate that RGS5 acts as a hypoxia-responsive protein in human brain pericytes that is regulated independent of hypoxia inducible factor-1α (HIF-1α), rapidly stabilized under hypoxia, but degraded under normoxic conditions. We show that RGS5 expression desensitizes pericytes to signalling of platelet-derived growth factor-BB (PDGFBB) and sphingosine 1-phosphate (S1P), and blocks chemokinesis or chemotaxis induced by these factors. Our data imply a role for RGS5 in antagonizing pericyte recruitment and retention to blood vessels during hypoxia and support RGS5 as a target in counteracting vessel leakage under pathological hypoxic conditions.

This article has an associated First Person interview with the first author of the paper.

## Teaser

**Summary:** Hypoxia leads to rapid stabilization of RGS5 in human brain pericytes, which counteracts signals essential for pericyte recruitment and retention.

## INTRODUCTION

Oxygen is fundamental for the survival and normal function of most multicellular organisms. Therefore, specific cellular mechanisms have evolved to sense and respond to hypoxia so that oxygen homeostasis is maintained within tissues and organs. The brain consumes ∼20% of the total available oxygen at rest, and, as a result, the brain vasculature has adapted to meet its high oxygen and energy demand, resulting in a 400 mile-long capillary network (Cipolla, 2009). Pericytes are perivascular cells that line the entire microvasculature of the brain and one of the key cell types that maintain blood-brain barrier (BBB) integrity and regulate the formation of new blood vessels (Armulik et al., 2010; Eilken et al., 2017). Due to their unique position at the blood-brain interface, pericytes are known to be one of the first responders to hypoxia (Duz et al., 2007; Gonul et al., 2002). During vascular development, pericytes are essential for vessel stabilization and maintenance (Daneman et al., 2010; Hellstrom et al., 1999), whereas in adulthood and under pathological conditions, they play an active role in modulating angiogenesis and maintaining the BBB integrity (Armulik et al., 2010; Kang et al., 2019; Ozerdem and Stallcup, 2003). These processes are often initiated as an adaptive response to hypoxic environments and are associated with the expression of regulator of G-protein signalling 5 (RGS5) (Mitchell et al., 2008), a protein that in the brain is exclusively expressed by vascular mural cells [pericytes and smooth muscle cells (SMCs)] (Bondjers et al., 2003; Cho et al., 2003).

RGS5 belongs to the R4 subfamily of RGS proteins and is a cytoplasmic protein that acts as a negative regulator of G-protein-coupled receptors (GPCRs) (De Vries et al., 2000). Recent discoveries have also highlighted non-GPCR-related intracellular targets, where RGS5 exerts a regulatory role (Hauser et al., 2018; Wang et al., 2021). RGS5 expression in pericytes in the adult brain is usually low under physiological conditions. Interestingly, however, RGS5 increases dramatically in pathological hypoxia (e.g. in tumours or ischaemic stroke), where it is associated with pericyte detachment and migration from the capillaries into the brain parenchyma, resulting in BBB leakage (Hamzah et al., 2008; Ozen et al., 2014, 2018; Roth et al., 2019). Those observations suggest that RGS5 is a prominent protein in the process of vascular remodelling and appears to play a regulatory role in the association of pericytes to the microvasculature, although the underlying mechanisms are not known.

Pericyte detachment from the microvasculature is a necessary process during vascular remodelling, allowing endothelial sprouting before subsequent initiation of pericyte migration and recruitment for stabilization of newly formed vessels (Kamouchi et al., 2012). The recruitment and retention of pericytes to the microvasculature is actively modulated by chemotactic factors secreted by endothelial cells, such as platelet derived growth factor-BB (PDGFBB) and sphingosine 1-phosphate (S1P) (Aguilera and Brekken, 2014; Jain, 2003; Karakiulakis et al., 2007; Renner et al., 2003; Wittko-Schneider et al., 2014). Previous studies on targeted deletion of PDGFBB and S1P signalling demonstrate an increase in pericyte dissociation from the vascular wall and lack of pericyte recruitment (Kono et al., 2004; Lindblom et al., 2003). However, whether the detachment or lack of recruitment of pericytes from microvessels in pathological hypoxia is related to disruption of pericyte chemotactic cues is unclear. The previous observations led us to hypothesize that the expression of RGS5 in hypoxic pericytes may play a regulatory role in PDGFBB- or S1P-induced pericyte recruitment and migration.

Here, we provide a detailed analysis of the regulation and the functional role of endogenous RGS5 expression in human brain pericytes under pathological hypoxic conditions. We show that RGS5 acts as a hypoxia-responsive protein in pericytes that is regulated independent of hypoxia inducible factor-1α (HIF-1α) and stabilized under hypoxia, whereas it is rapidly degraded under normal oxygen conditions *in vitro*. We examine the function of RGS5 under hypoxic conditions using siRNA silencing and characterize proliferation, apoptosis, chemokinetic and chemotactic migration of brain pericytes as well as downstream mitogen-activated protein kinase (MAPK) signalling after PDGF receptor-β (PDGFRβ) and S1P receptor (S1PR) stimulation. We demonstrate that RGS5 expression under hypoxia reduces PDGFRβ phosphorylation and desensitizes pericytes to the chemotactic and chemokinetic response induced by PDGFBB, while this effect is not observed under normoxic conditions. In addition, RGS5 inhibits the chemokinetic migratory response of pericytes to S1P. These findings suggest a role for RGS5 in antagonizing pericyte retention and/or recruitment to endothelial cells during hypoxia and vascular remodelling.

## RESULTS

### RGS5 protein levels in brain pericytes are stabilized under hypoxia

**Fig. 1.**
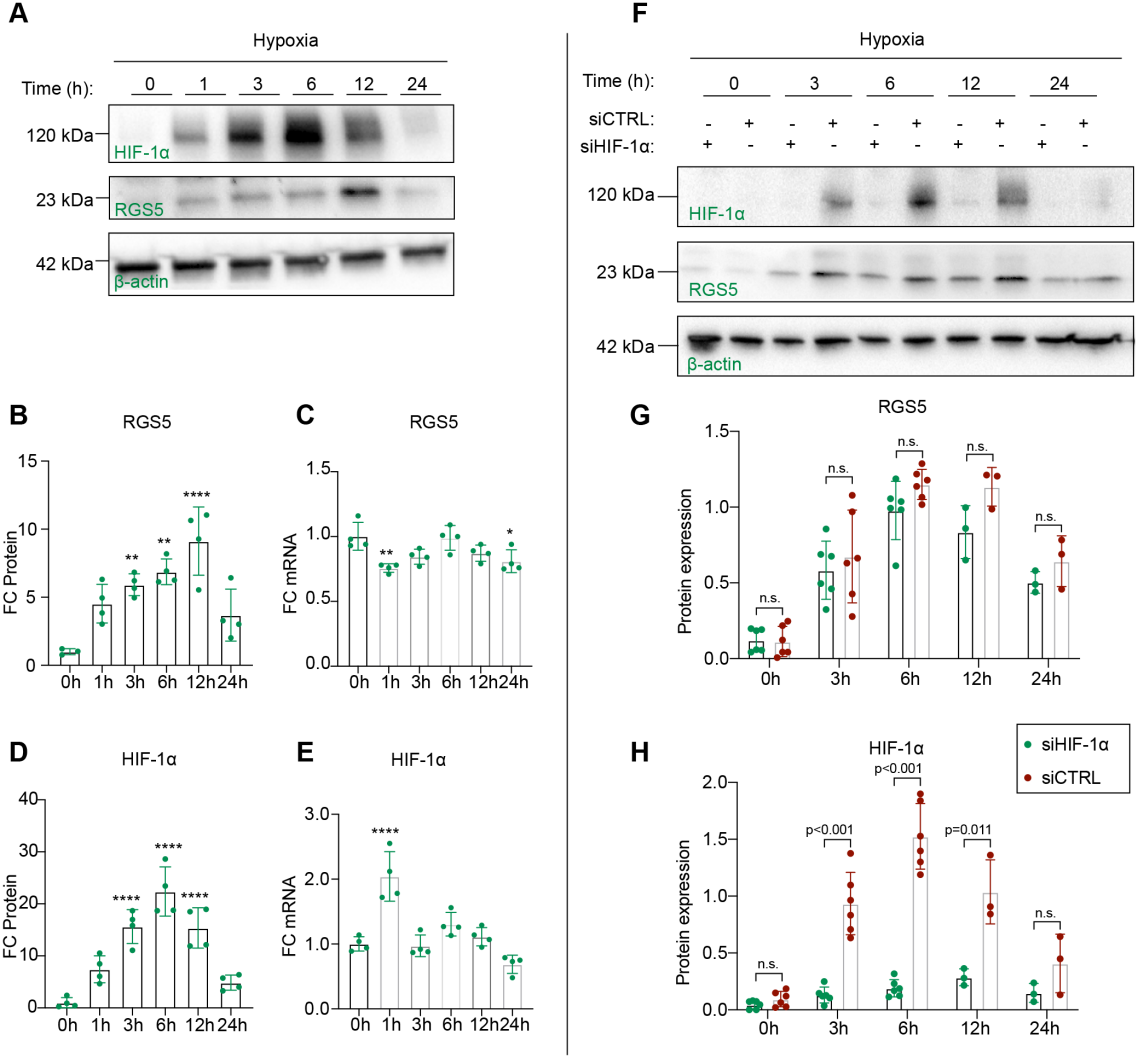
**RGS5 is induced under hypoxia but not regulated by HIF-1α.** (A) Human brain pericytes were exposed to a set time course of hypoxia (O_2_<1%) for 0, 1, 3, 6, 12 and 24 h. Representative western blot of RGS5, HIF-1α and β-actin is shown. (B,D) Quantification of relative protein levels of RGS5 and HIF-1α obtained from western blot normalized to β-actin as a house-keeping control. Data are presented as mean±s.d. Statistical significance refers to testing with 0 h of hypoxia as control group using one-way ANOVA with Tukey's multiple comparisons. For RGS5 timeline (B), **P=0.0071 for 3 h, **P=0.001 for 6 h and ****P<0.0001 for 12 h. For HIF-1α timeline (D), all significant time points had *P*<0.0001. Sample size is *n*=3 or 4. (C,E) Relative mRNA quantification of *RGS5* and *HIF1A* was normalized to β-2 microglobulin (*β2M*), and fold change (FC) was calculated from pericytes under normal oxygen conditions as control group. Statistical significance refers to testing with 0 h of hypoxia as control group. For *RGS5* timeline (C), **P=0.0036 for 1 h and *P=0.027 at 24 h. For *HIF1A* timeline (E), ****P<0.0001 for 1 h. Data are presented as mean±s.d. Statistical analysis was performed using one-way ANOVA with Tukey's multiple comparisons, *n*=4. (F) Representative western blot of HIF-1α, RGS5 and β-actin over a set time course of hypoxia (O_2_<1%) for 0, 3, 6, 12 and 24 h. (G,H) Human brain pericytes were transfected with specific siRNA targeting HIF-1α or scramble control siRNA and exposed to hypoxia (O_2_<1%) for 0, 3, 6, 12 and 24 h. Protein expression of RGS5 (G) and HIF-1α (H) was measured with western blotting. Data are presented as mean±s.d. Statistical analysis was performed using unpaired multiple *t*-tests, *n*=3 or 6. n.s., not significant.

Based on our previous observations of increased RGS5 expression in brain pericytes in hypoxic/ischaemic pathology *in vivo* (Ozen et al., 2014, 2018; Roth et al., 2019), we hypothesized that RGS5 is mainly present and has its primary function in hypoxic environments. Confirming our hypothesis, we found that endogenous RGS5 protein expression was extremely low in normoxia (Fig. 1A,B).

In contrast, when we exposed pericytes to hypoxia, we detected a time-dependent induction in RGS5 protein levels. We observed a ∼5-fold increase in RGS5 as early as after 1 h, which gradually increased and reached its peak at 12 h before returning towards baseline at 24 h, confirming that RGS5 is hypoxia inducible (Fig. 1B). Similarly, hypoxia increased the protein levels of HIF-1α, a known hypoxia-inducible transcription factor, in a time-dependent manner, showing a continuous increase and peak at 6 h before returning towards baseline at 24 h (Fig. 1D). However, *RGS5* mRNA expression remained relatively constant, displaying slight but even significantly lower levels of mRNA at both 1 and 24 h of hypoxia (Fig. 1C). Similarly, *HIF1A* mRNA remained constant except for a significant increase at 1 h of hypoxia (Fig. 1E), indicating that both hypoxia-inducible proteins are regulated primarily post-translationally.

### Hypoxia-induced RGS5 expression in human brain pericytes is independent of HIF-1α

Many of the so far discovered adaptive cellular responses to hypoxia are linked to transcriptional activation by HIFs (Majmundar et al., 2010). This has also been claimed for RGS5; however, this study was performed in human-umbilical-vein endothelial cells forced to overexpress RGS5 (Jin et al., 2009).

To directly evaluate whether the transcription factor HIF-1α also regulates RGS5 expression in pericytes, we used siRNA to silence the expression of *HIF1A* and measured protein levels of HIF-1α and RGS5 under hypoxia. *HIF1A* siRNA significantly downregulated HIF-1α protein expression, confirming the efficiency of the siRNA silencing (Fig. 1F,H). Consistent with the relatively constant levels of *RGS5* mRNA shown previously, siRNA knockdown targeting *HIF1A* did not lead to a significant change in RGS5 protein levels at any of the time points of 0, 3, 6, 12 or 24 h of hypoxia, confirming that the hypoxic induction of RGS5 is not regulated by HIF-1α-initiated transcription (Fig. 1F-H).

Our findings suggest that RGS5 functions as a hypoxia-responsive protein in brain pericytes and that its protein induction following hypoxia is controlled outside of the hypoxia-regulated transcriptional network and is independent of HIF1α.

### RGS5 attenuates PDGFBB-induced chemotaxis of pericytes in hypoxia

**Fig. 2.**
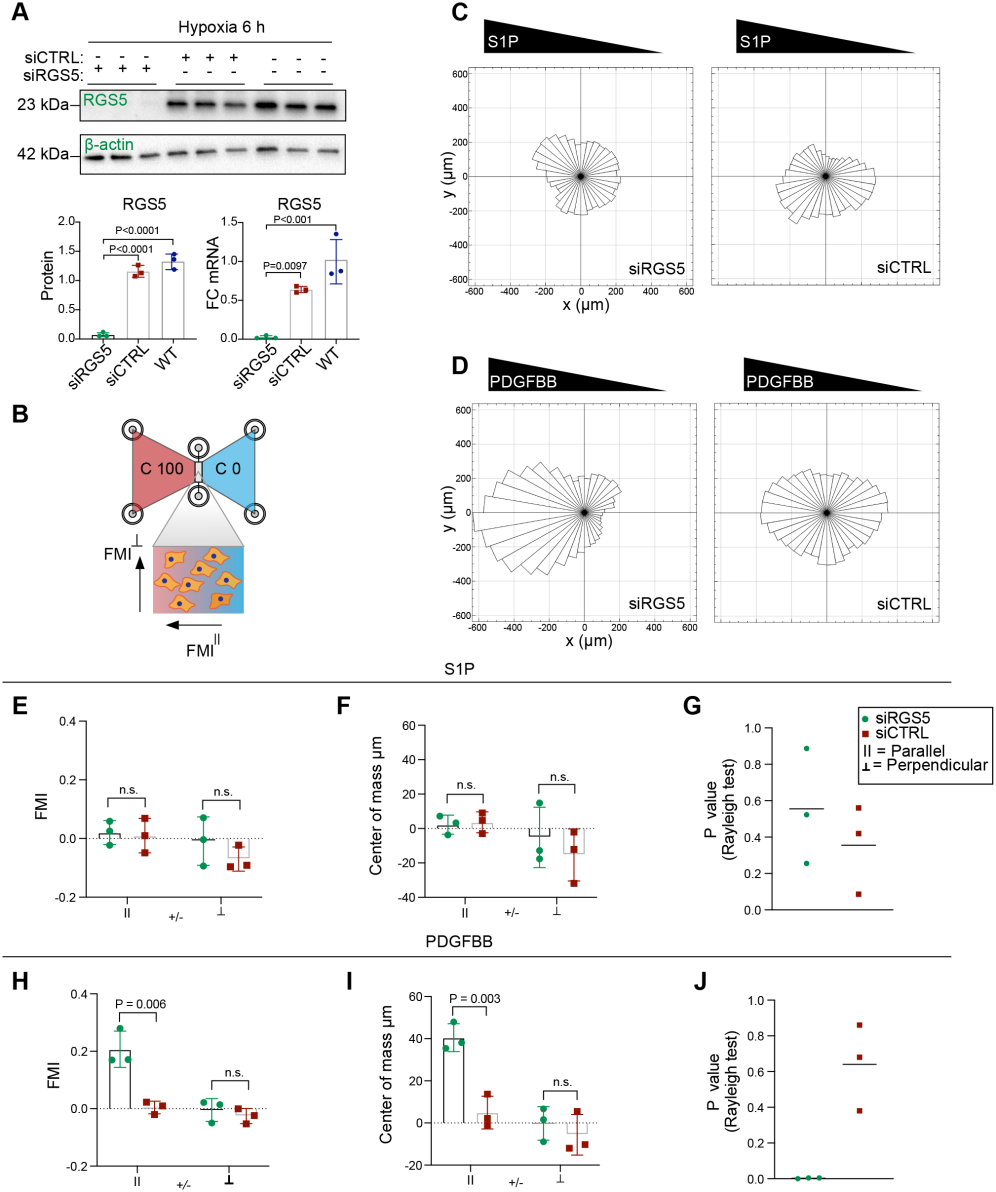
**RGS5 inhibits PDGFBB-induced chemotaxis under hypoxia.** (A) RGS5 knockdown was verified at the mRNA and protein level in human brain pericytes after siRNA treatment targeting RGS5 compared to control scrambled siRNA and WT using western blotting and real-time qPCR after 6 h of hypoxia. Data are presented as mean±s.d. Statistical analysis was performed using ordinary one-way ANOVA with Tukey's multiple comparisons, *n*=3. (B) Illustration of the microfluidic chambers used for the chemotaxis experiments with a stable linear gradient from concentration (C) of 100% to 0%. Forward migration index (FMI) is illustrated as parallel (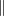) or perpendicular (⊥) to the gradient. (C) Role of RGS5 in the chemotactic response to chemical gradients of S1P (1 μM) imaged during 1-23 h of hypoxia. The sum of 120 individual cellular trajectories from siRGS5 and siCTRL are shown collectively illustrated in a rose plot, *n*=3. (D) Role of RGS5 in the chemotactic response to chemical gradients of PDGFBB (50 ng/ml) imaged during 1-23 h of hypoxia. The sum of 160 individual cellular trajectories from siRGS5 and siCTRL are shown collectively illustrated in a rose plot, *n*=3. (E-J) The FMI (E,H), centre of mass (F,I) and circular distribution of cell trajectories (G,J) were calculated to assess the chemotactic migration in response to S1P (1 μM) or PDGFBB (50 ng/ml). The data are presented as mean±s.d. Statistical analysis was performed using multiple *t*-tests for FMI and centre of mass, and Rayleigh test was computed for the evaluation of circular distribution uniformity of cell trajectory endpoints. *P*-values are indicated.

To study the role of RGS5 in human brain pericytes under hypoxia, we utilized siRNA silencing of RGS5 (siRGS5) and compared the response to pericytes treated with scrambled control siRNA (siCTRL). Successful knockdown of RGS5 was verified for both mRNA and protein expression (Fig. 2A). Scrambled siCTRL showed similar levels of RGS5 when compared to wild-type (WT) pericytes (Fig. 2A).

The recruitment and retention of pericytes to newly formed vessels are necessary mechanisms for vessel maturation and rely on specific chemotactic factors secreted by endothelial cells, such as S1P and PDGFBB (Aguilera and Brekken, 2014; Jain, 2003; Raza et al., 2010).

Using a microfluidic migration chamber with a stable linear concentration gradient, we examined the effect of RGS5 on S1P-induced chemotaxis of pericytes under hypoxia (Fig. 2B). We performed real-time quantitative PCR (qPCR) to analyse which of the five different S1P receptors was expressed in the pericyte cell line used in this study. We observed a clear expression of *S1PR2* and *S1PR3* mRNA in both normoxic and hypoxic environments that was not affected by siRGS5. However, upon hypoxic exposure for 24 h, we observed a ∼ 2-3-fold decrease in both S1PR2 and S1PR3 in pericytes. We detected a slight expression of S1PR1 but complete lack of S1PR4 and S1PR5 expression in pericytes (Fig. S1). There was no clear chemotactic effect after S1P treatment for either the forward migration index (FMI) or centre of mass distribution, nor an effect on circular distribution homogeneity of cellular trajectory endpoints, where pericytes migrated randomly independent of RGS5 expression and the S1P gradient (Fig. 2C,E-G). To verify that the lack of chemotactic migration in response to S1P was not a consequence of hypoxic exposure, we conducted the same experiment under normoxic conditions. Likewise, S1P did not induce a clear effect of chemotaxis in both the siRGS5- and siCTRL-treated pericytes (Fig. S2A, B)

Next, we investigated whether RGS5 regulates the chemotactic response to PDGFBB, a strong chemoattractant factor for pericytes during vascular remodelling. Here, knockdown of RGS5 resulted in a significantly increased sensitivity of pericytes to PDGFBB, while siCTRL pericytes expressing RGS5 instead exhibited more random migration (Fig. 2D). The FMI parallel (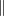) to the gradient was higher and showed a more consistent degree of migration towards the source of the gradient in siRGS5 compared to siCTRL pericytes (Fig. 2H). Similarly, the centre of mass representing the average of all single-cell endpoints was shifted towards the PDGFBB gradient to a significantly higher degree in siRGS5 compared to siCTRL pericytes (Fig. 2I). The Rayleigh test showed a significant inhomogeneous distribution of cellular endpoints in siRGS5 compared to uniform circular distribution for siCTRL pericytes (Fig. 2J), indicating that expression of RGS5 desensitizes pericytes to the chemotactic cues of PDGFBB. Furthermore, we verified these findings by conducting the same experiment under normoxic conditions in which RGS5 is degraded. PDGFBB induced a strong chemotactic effect in both siRGS5- and siCTRL-treated pericytes, suggesting that the inhibitory effect of RGS5 on PDGFBB chemotaxis is hypoxia dependent (Fig. S2C,D). These results indicate that the knockdown of RGS5 in hypoxia rescues the PDGFBB-induced chemotaxis to ∼ 50% compared to normoxia.

### RGS5 reduces PDGFBB- and S1P-induced chemokinesis in hypoxia

**Fig. 3.**
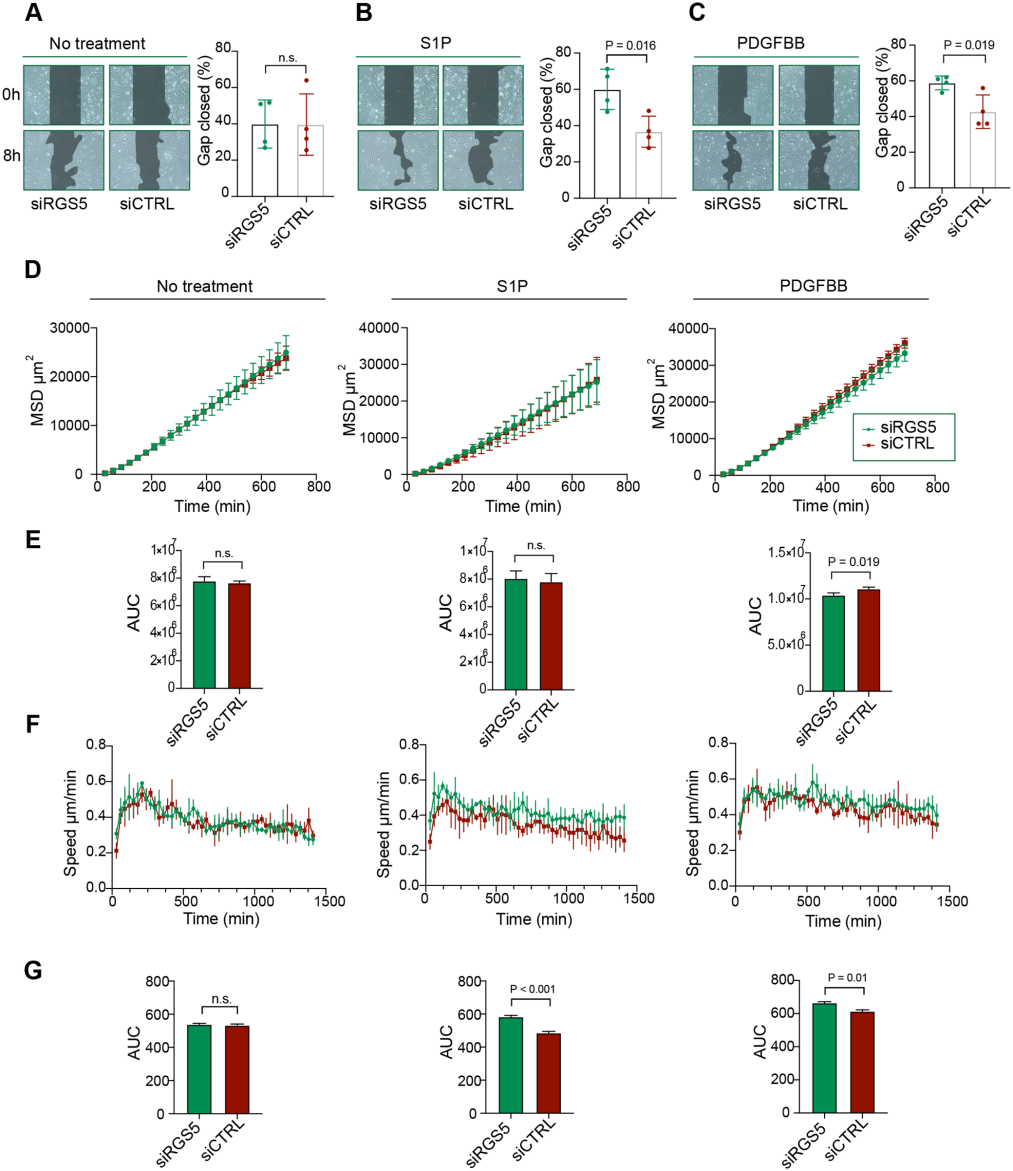
**RGS5 reduces the efficiency of pericyte migration under hypoxia in response to PDGFBB and S1P.** (A-C) Representative images for wound-healing assay of non-treated pericytes (A) or after exposure to S1P (1 μM) (B) or PDGFBB (5 ng/ml) (C). Images represent the beginning time (0 h) or endpoint (8 h) of the experiment under hypoxia. Quantification of relative collective migration as percentage ratio of wound area at time 8 h compared to wound area at time 0 h expressed as % gap closed. Data are presented as mean±s.d. Statistical analysis was performed using unpaired Student's *t*-test, *n*=4. (D) Single cells of siRGS5 and siCTRL were tracked for the duration of 24 h with 30 min frame intervals under hypoxia. Mean square displacement coefficients (MSD) as a function of time over 690 min with 30 min intervals and represented in a linear plot. (E) The area under the curve (AUC) was calculated from the MSD curves. (F) The instantaneous speed was calculated for each time point during the time lapse. (G) The AUC of the instantaneous speed data was used for hypothesis testing. Pericytes were either non-treated or treated with S1P (1 μM) or PDGFBB (5 ng/ml), respectively. Data are presented as mean±s.d. Statistical analysis was performed using unpaired Student's *t*-test, *n*=3.

Next, we investigated if RGS5 regulated the efficiency of pericyte motility at a basal level or elicited a change in S1P- or PDGFBB-induced chemokinesis. To determine the effect of RGS5 on collective pericyte migration, we performed a wound-healing scratch assay for the duration of 8 h under hypoxia. RGS5 expression did not impact pericyte motility under non-stimulated conditions (siRGS5=39.9±13%; siCTRL=39.6±17%) (Fig. 3A). However, knockdown of RGS5 in pericytes stimulated with S1P showed a significant increase in percentage of wound closure compared to siCTRL (siRGS5=60.0±11%; siCTRL=36.7±8%) (Fig. 3B). The addition of PDGFBB also resulted in a slight, but significant, increase in cellular motility in siRGS5 compared to siCTRL pericytes (siRGS5=58.8±4%; siCTRL=42.7±9%) (Fig. 3C).

As the wound-healing assay is limited due to the rapid gap closure, cell-cell interactions and inconsistencies in scratch diameters, we decided to validate our findings using long-term single-cell migration analysis for 24 h under hypoxia. We calculated the mean square displacement (MSD) to evaluate the overall efficiency of migration as it considers both cellular speed and direction. Here, we observed similar MSD values between non-stimulated siRGS5 compared to siCTRL pericytes under hypoxic conditions (Fig. 3D,E). Likewise, S1P treatment did not reveal any difference in MSD after long-term single-cell migration under hypoxia, and only a slight difference after PDGFBB treatment between siRGS5 and siCTRL pericytes (Fig. 3D,E). Next, we assessed the instantaneous speed of migrating pericytes for every 30 min time interval. Despite a time-dependent trend towards a decrease in the speed of migration during 24 h of hypoxia, pericytes remained migratory throughout the time period. RGS5 did not influence the instantaneous speed of non-treated pericytes (Fig. 3F,G). However, siRGS5 compared to siCTRL pericytes migrated slightly faster after S1P and PDGFBB treatment shown from the instantaneous speed and the area under the curve (AUC) evaluation, although the effect size observed is small and may not confer any functional difference. Pericytes treated with *RGS5* siRNA showed no changes in MSD values after S1P treatment and even significantly lower MSD values after PDGFBB treatment, although with an effect size of only ∼6% despite having higher instantaneous speed after S1P and PDGFBB treatment (Fig. 3E-G). Since MSD takes directionality of cellular migration into account, our results suggest that siRGS5 pericytes have more meandering cellular trajectories compared to siCTRL pericytes but increased speed of migration after stimulation with S1P or PDGFBB, in absence of a concentration gradient. This is due to the slope of the MSD curves being dependent on the persistence of the random walk of the cellular trajectory and cellular speed of migration. As the persistence of directionality diminishes, it reduces the slope of the MSD coefficients. Thus, the siRGS5 pericytes migrating at faster speed when treated with PDGFBB or S1P but exhibiting similar or even slightly lower MSD curves suggest that they also exhibit more meandering migratory trajectories.

Cellular geometry is intricately linked to cellular motility. The balance between retraction and expansion of cellular protrusions alters the cellular shape of migrating cells (Chen et al., 2013). To assess if RGS5 regulated cellular shape, we measured cellular solidity as well as the area of the cell body after 24 h of hypoxia. However, no significant differences were observed between siRGS5 and siCTRL pericytes regardless of stimuli (Fig. S3).

In summary, our findings demonstrate that RGS5 reduces the chemokinetic effect of S1P and PDGFBB on pericytes in hypoxia during collective and long-term single-cell migration.

### RGS5 inhibits S1P-induced MAPK signalling and PDGFRβ-phosphorylation in hypoxia

**Fig. 4.**
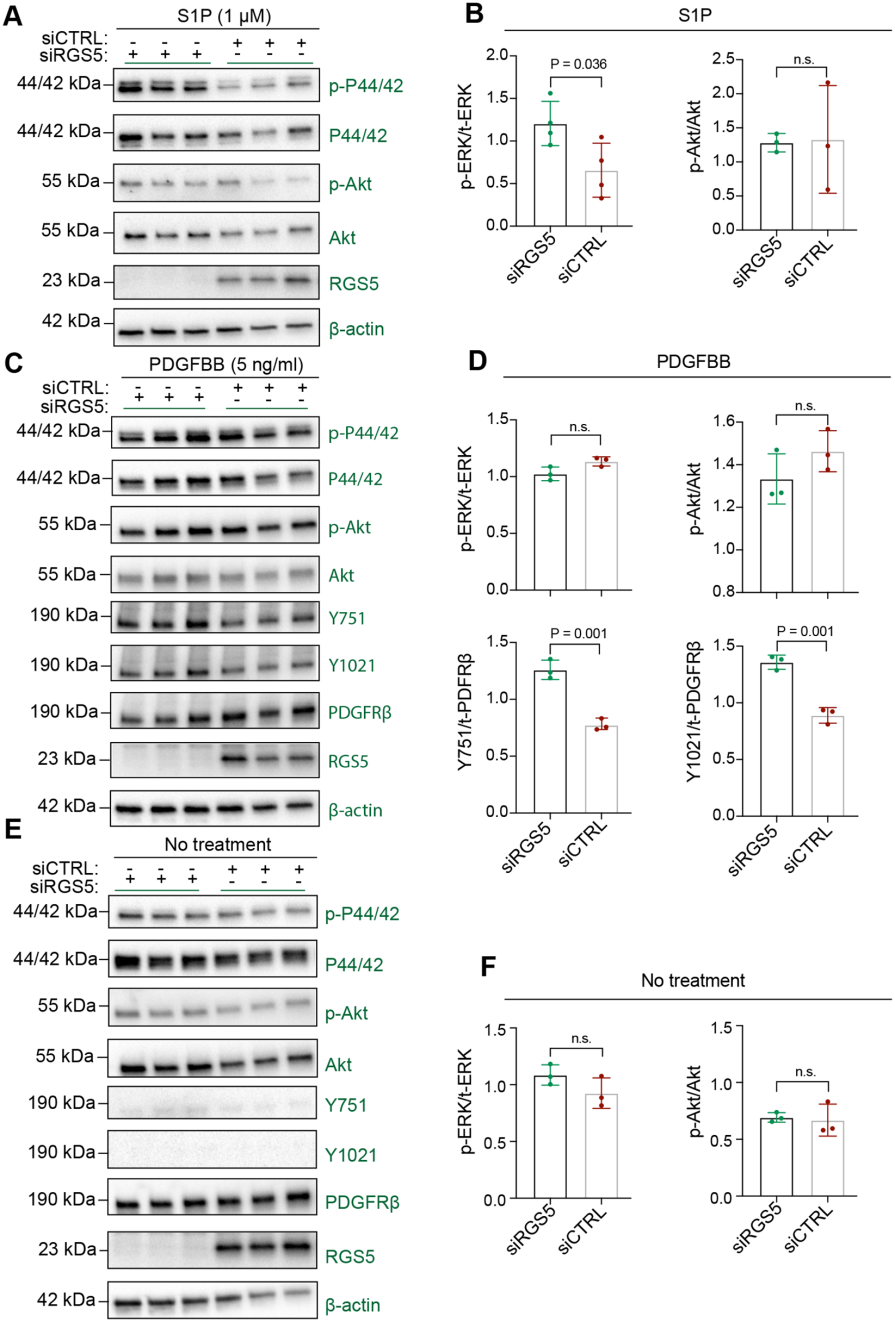
**RGS5 regulates S1P-induced MAPK activation and PDGFRβ phosphorylation.** (A,B) Western blot analysis of p-ERK/ERK, p-AKT/AKT signalling in siRGS5 and siCTRL pericytes. Cells were exposed to 8 h of hypoxia before S1P (1 μM) treatment for 5 min. β-actin was used as a house-keeping control and RGS5 knockdown was verified. One independent replicate was blotted on a separate membrane, while three independent replicates are illustrated in the figure originating from the same membrane. (C,D) Western blot analysis of p-ERK/ERK, p-AKT/AK, PDGFRβ-p-Y1021, -p-Y751 and total PDGFRβ in siRGS5 compared to siCTRL pericytes. Cells were exposed to 8 h of hypoxia before PDGFBB (5 ng/ml) treatment for 5 min. β-actin was used as a house-keeping control and RGS5 knockdown was verified. (E,F) Western blot analysis of p-ERK/ERK, p-AKT/AKT, p-Y1021, p-Y751 and total PDGFRβ in siRGS5 compared to siCTRL pericytes. Non-treated cells were exposed to 8 h of hypoxia before extraction. β-actin was used as a house-keeping control and RGS5 knockdown was verified. The data are presented as mean±s.d. Statistical analysis was performed using unpaired Student's *t*-test, *n*=3-4. *P*-values are indicated.

Next, we investigated the effect of RGS5 on intracellular signalling targets and PDGFRβ phosphorylation. MAPK signalling via extracellular-regulated kinase (ERK1/2) and protein kinase B (AKT) phosphorylation is important for cellular proliferation and migration as ERK1/2 and AKT are downstream signalling targets that modify e.g. cell cycle S-phase entry and cytoskeletal polarity (Bonacchi et al., 2001; Kluk and Hla, 2002; Mebratu and Tesfaigzi, 2009). As MAPK signalling occurs downstream of both G-protein S1PRs and PDGFRβ, we wanted to assess the potential role of RGS5 as a regulator of their activation in brain pericytes under hypoxic conditions (Cho et al., 2003; Gunaje et al., 2011). Therefore, we treated siRGS5 or siCTRL pericytes with PDGFBB or S1P for 5 min after the cells had been exposed to 8 h of hypoxia. We observed a significant increase in phosphorylated (p)-ERK in siRGS5 compared to siCTRL pericytes upon S1P stimulation but no significant effect on p-AKT (Fig. 4A,B). However, these results display a large standard deviation and should be interpreted with caution. PDGFBB-initiated PDGFRβ phosphorylation induced a significantly higher degree of phosphorylation of both Y751 and Y1021 tyrosine residues on the PDGFRβ intracellular domain in siRGS5 compared to siCTRL pericytes (Fig. 4C). However, no significant difference was observed in p-ERK or p-AKT signalling between siRGS5 and siCTRL pericytes after PDGFBB treatment (Fig. 4C,D). Non-treated pericytes showed no significant difference in basal p-ERK nor p-AKT activation between siRGS5 and siCTRL pericytes. Furthermore, non-treated cells elicited undetectable levels of both Y1021 and Y751 of p-PDGFRβ regardless of RGS5 expression under hypoxia (Fig. 4E,F). In addition, we evaluated the signalling targets in normoxia, where we did not detect a significant difference between siRGS5 and siCTRL cells, after S1P or PDGFBB treatment or under non-treated conditions (Fig. S4A-E). Our results displayed a trend for increased p-ERK after both PDGFBB and S1P stimuli, but this was not significant compared to non-treated conditions (Fig. S4B). However, PDGFBB treatment had a clear effect on AKT and PDGFRβ phosphorylation (Fig. S4C-E).

The importance of ERK phosphorylation in cell cycle entry throughout G0/G1 to facilitate S-phase progression is well documented (Mebratu and Tesfaigzi, 2009). Likewise, PDGFRβ activation is known to induce pericyte proliferation and survival (Payne et al., 2021; Renner et al., 2003). As we had shown that RGS5 interferes with p-ERK and p-PDGFRβ after S1P or PDGFBB treatment, respectively, we next investigated whether RGS5 has the potential of regulating pericyte proliferation or apoptosis.

### RGS5 does not regulate pericyte proliferation or apoptosis

Even though hypoxia induces cell cycle arrest in most cells, certain cell types that reside in the hypoxic niche, including endothelial cells, SMCs and pericytes, may retain their proliferative capacity during e.g. hypoxia-induced angiogenesis (Hubbi and Semenza, 2015; Namiki et al., 1995; Phillips et al., 1995; Lannér et al., 2005). We have previously observed that loss of RGS5 *in vivo* resulted in increased numbers of pericytes in the infarct core 24 h after ischaemic stroke (Ozen et al., 2018). We hypothesized that this effect could, at least in part, be the consequence of RGS5 regulating pericyte proliferation or apoptosis.

**Fig. 5.**
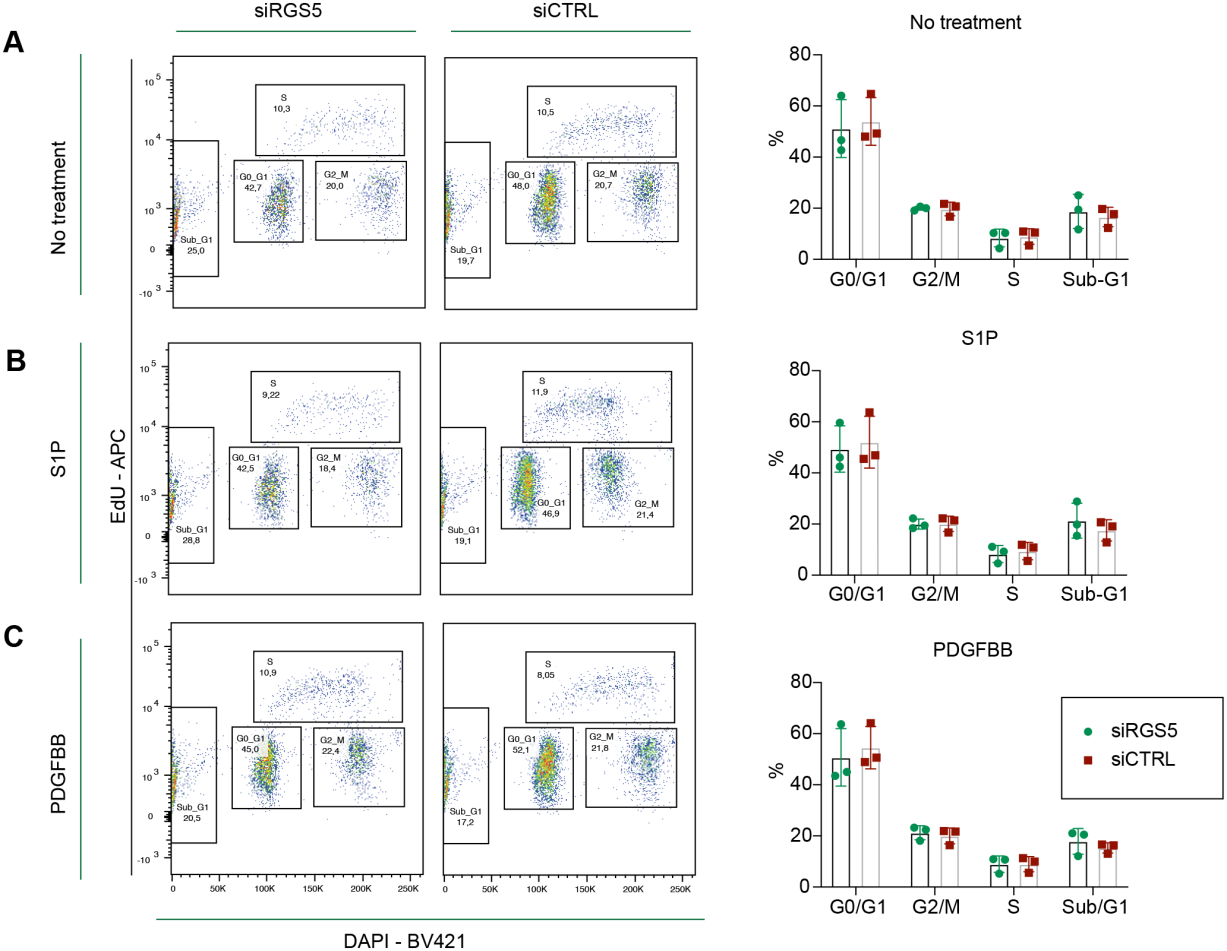
**RGS5 does not regulate human brain pericyte proliferation under hypoxia. (**A-C) EdU incorporation assay was performed to measure pericyte proliferation activity in siRGS5 and siCTRL pericytes. All experimental groups were exposed to hypoxia for 8 h and treated with EdU during the last hour of incubation. The percentage of EdU (APC-A)- and DAPI (BV421)-positive cells was used to measure pericytes in S-phase or G0/G1 and G2/M and sub-G1 with flow cytometry. Non-treated (A) or pericytes treated with S1P (1 μM) (B) or PDGFBB (5 ng/ml) (C) after 1 h in hypoxia were analysed. The data are presented as mean±s.d., *n*=3.

Therefore, we analysed the different stages of the cell cycle using 5-ethynyl-2′-deoxyuridine (EdU) incorporation and nuclear staining by flow cytometry. Despite that pericytes retained proliferative activity after 8 h of hypoxia, knockdown of RGS5 did not lead to significant changes in cell cycle progression of G0/G1, S, G2 or mitosis compared to siCTRL pericytes under non-stimulated conditions (Fig. 5A). Furthermore, S1P or PDGFBB treatment resulted in similar pericyte proliferation profiles to non-stimulated conditions regardless of RGS5 expression (Fig. 5A-C).

To determine whether loss of RGS5 may instead affect apoptosis in pericytes, we also evaluated the sub-G1 peak corresponding to nuclear fragmentation of apoptotic cells. We did not observe any significant difference between siRGS5 and siCTRL pericytes, with or without PDGFBB or S1P stimuli (Fig. 5A-C).

Our results indicate that pericytes’ propensity to complete cellular division or undergo apoptosis after PDGFBB or S1P treatment under hypoxic conditions remained unchanged compared to that of non-stimulated cells and is not affected by RGS5 expression.

## DISCUSSION

Understanding the molecular mechanisms governing cellular oxygen sensing is necessary to discern how cells respond to hypoxia and the pathophysiological implications thereof. It also allows the exploration of novel targets to modulate these processes. When hypoxia occurs in the brain, pericytes that are uniquely located at the blood-brain interface are one of the first responders and express high levels of RGS5 (Duz et al., 2007; Ganss, 2015; Gonul et al., 2002; Ozen et al., 2018; Roth et al., 2019).

Here, we show that RGS5 acts as a hypoxia-responsive protein in brain pericytes. We demonstrate that endogenous RGS5 protein in brain pericytes *in vitro* is present and stabilized under hypoxic conditions. Previously, RGS proteins have often been studied under normoxic conditions in cells with otherwise little endogenous expression of RGS5 using overexpression models (Ganss, 2015; Jin et al., 2009). Whilst studies using vector expression of RGS5 may be a helpful tool to investigate molecular mechanisms regulated by RGS5, they introduce highly enriched protein levels and in conditions in which it may be physiologically irrelevant, such as in normoxia. To our knowledge, this is the first time that RGS5 is identified as a hypoxia responder in brain pericytes in which RGS5 protein expression is shown to be dependent on a hypoxic environment. Our results are in line with recent evidence demonstrating that RGS5 is a subject of the NO/O_2_-dependent Arg/N-degron pathway and undergoes rapid proteolysis in the presence of oxygen due to its N-terminal configuration. In the case of RGS5, when oxygen is available, the cysteine residue at the N-terminal is oxidized and recognized by arginyl-tRNA-protein transferase, which transfers arginine to the cysteine residue and primes RGS5 for ubiquitin ligation and degradation (Lee et al., 2005; Masson et al., 2019; Ganss, 2015). Thus, in normoxia, GPCR and other signalling mechanisms can be maintained due to the absence of RGS5 that would otherwise inhibit receptor downstream activation. It has been postulated that the conditionally short half-life of RGS5 under normoxia is a cellular mechanism to ensure maximal responsiveness to environmental changes like hypoxia (Lee et al., 2012). Thus, in a hypoxic environment, the N-terminal oxidation of RGS5 is inhibited and the degradation process stopped. Our results demonstrate that the induction of RGS5 in hypoxia is exclusively regulated post-translationally, where RGS5 stabilization is a rapid real-time response to hypoxia and independent of HIF-1α-mediated transcriptional activation. On a physiological level, it is likely that the direct post-translational stabilization of RGS5 leads to faster adaptations to hypoxia than the transcriptional responses transduced by HIF-1α in brain pericytes.

As pericytes are crucial for vascular stability and remodelling during vasculogenesis and angiogenesis, we investigated the effect of RGS5 on S1P and PDGFBB signalling, known to be key angiogenic factors that regulate and coordinate pericyte recruitment, retention and proliferation.

Pericytes typically express S1PR1, S1PR2 and S1PR3 (He et al., 2018; Uemura et al., 2020; Vanlandewijck et al., 2018). Previous studies support the notion of S1P signalling being important for mural cell association to blood vessels (Cartier et al., 2020; Kono et al., 2004). S1P signalling also has chemotactic responses in various cell types including macrophages and SMCs (Duru et al., 2012; Keul et al., 2011), but the contribution of S1P signalling in pericytes during vascular formation and pericyte recruitment is not clear. We observed expression of S1PR1-S1PR3 in our pericyte cell line, which can signal via the G_i_ or G_q_ GPCR pathway but can induce opposing cellular effects. For example, overexpressing S1PR1 induces enhancement of mural cell coverage, while knocking out S1PR2 yields similar results (Cartier et al., 2020). Since RGS5 interacts with G_i_ and G_q_ signalling pathways we wanted to test our hypothesis that S1P signalling in pericytes may induce chemotaxis, where RGS5 could play a regulatory role. In line with these results, previous studies have shown a link between S1P signalling and induction of SMC migration (Böhm et al., 2013; Liu et al., 2018). Studies performed under normoxic conditions on human aortic SMCs overexpressing RGS5 or in rat aortic SMCs with endogenous RGS5 expression have suggested that RGS5 attenuates S1PR signalling (Cho et al., 2003; Gunaje et al., 2011). Here, we demonstrate that RGS5 significantly inhibits downstream phosphorylation of ERK but did not have a significant effect on p-AKT after S1P treatment in brain pericytes in the context of pathological hypoxia. This suggests that RGS5 may have a regulatory role in the intrinsic GTPase activity of the G-protein-coupled S1PRs. However, these results display a large standard deviation and should be interpreted with caution, as the role of RGS5 in MAPK signalling after S1P stimulation requires further investigation. Furthermore, we demonstrate that RGS5 elicits an inhibitory function in S1P-induced chemokinesis; however, S1P did not have a chemotactic effect on pericytes.

The initiation of vascular remodelling requires pericyte detachment from the vessel wall, allowing endothelial sprouting before pericytes are recruited back to the endothelial cell layer to achieve vessel maturation (Kamouchi et al., 2012). Pericytes thereby respond to the chemotactic cues of PDGFBB secreted by endothelial cells (Aguilera and Brekken, 2014). PDGFBB, once secreted, stays normally bound to the extracellular matrix because of its C-terminal retention motif (Ostman et al., 1991). Targeted deletion of the retention motif leads to partial dissociation of pericytes and cellular protrusions extending away from the vascular wall (Lindblom et al., 2003). This confirms that PDGFBB is not only necessary for pericyte recruitment but is also important for pericyte retention to the endothelium.

Interestingly, our results demonstrate that RGS5 reduces the sensitivity of pericytes to PDGFBB-induced chemokinesis and chemotaxis, thereby antagonizing recruitment and retention of pericytes to the endothelium under hypoxic conditions. These results are in line with our recent studies in an *in vivo* model of ischaemic stroke, showing that pericytes respond early to stroke and express RGS5 before they detach from the endothelial cell layer and migrate into the brain parenchyma, causing perivascular depletion of pericytes. Under these pathological conditions, this originally adaptive process of pericyte detachment during vascular remodelling leads to BBB breakdown, increased oedema and aggravation of neuronal cell death (Ozen et al., 2014, 2018). In turn, we and others have previously shown that when RGS5 is knocked out in models of ischaemic stroke (Ozen et al., 2018; Roth et al., 2019) or tumours (Hamzah et al., 2008), pericyte coverage of the vascular wall and vessel integrity are preserved and vascular leakage is substantially reduced. Thus, our present data demonstrate that expression of RGS5 is a possible mechanism for pericyte detachment or lack of pericyte recruitment as an adaptive response to hypoxic conditions by counteracting PDGFBB-induced chemotactic cues. This is further demonstrated by the strong PDGFBB chemotactic effect in normoxia regardless of siRGS5 or siCTRL treatment due to the constant degradation of RGS5 in the presence of oxygen. Interestingly, knockdown of RGS5 in hypoxia conserves ∼ 50% of the PDGFBB-induced chemotaxis; however, in the RGS5-expressing siCTRL pericytes, this effect was lost.

Even though the focus of this study is the regulation of RGS5 and its functional role in hypoxia, we also investigated some of the possible underlying mechanisms of the reduced responsiveness to PDGFBB under hypoxia. Specifically, we show that RGS5 expression is associated with inhibition of phosphorylation of the tyrosine intracellular domains Y751 and Y1021 of PDGFRβ upon PDGFBB stimulation. However, we did not detect any difference in phosphorylation of the downstream targets ERK or AKT after PDGFRβ activation, suggesting that PDGFBB-induced chemotaxis and chemokinesis are regulated via other downstream signalling trajectories or different mechanisms under hypoxia (Dubrac et al., 2018). Because PDGFRβ phosphorylation and dimerization after ligand binding is dependent on several scaffolding proteins to induce downstream signalling networks with the potential to induce pericyte migration (Dubrac et al., 2018; Aguilera and Brekken, 2014), further studies that e.g. evaluate the phospho-proteomic profile regulated by RGS5 in response to PDGFBB are needed.

While RGS5 expression impairs pericyte migration and recruitment *in vitro*, pericyte proliferation remained unchanged, regardless of stimuli and RGS5 expression under hypoxia. Even under normal physiological conditions, the initiation of cellular division is a tightly controlled and a highly energy-consuming process that requires cellular sensing mechanisms to regulate and facilitate cell cycle progression. Our results show that pericytes instead favour a migratory phenotype over proliferation alterations under pathological hypoxia.

Hypoxia promotes the highly coordinated angiogenic or vasculogenic response. It is possible that the susceptibility of RGS5 stabilization/degradation to fluctuations in oxygen levels has a role in fine tuning the process of vascular remodelling. In vascular pathology like ischaemic stroke, tissue oxygenation is severely disturbed, leading to severe hypoxia and likely fast stabilization kinetics of RGS5, where its effects remain mostly unexplored. It is conceivable that the stabilization of RGS5 under prolonged hypoxia may lead to a dysregulation in pericyte signalling during vascular remodelling and possibly contributes to perivascular pericyte loss in hypoxic/ischaemic brain pathology.

In summary, we demonstrate that RGS5 is a hypoxia-responsive protein in human brain pericytes that is regulated independent of HIF-1α and allows a rapid real-time response to hypoxia able to decouple pericytes from specific extracellular signals related to pericyte recruitment and retention to the vasculature. Thus, RGS5 may constitute a target in pericytes to modulate unbalanced responses to hypoxia under pathological situations.

## MATERIALS AND METHODS

### Cell culture

Primary human brain pericytes isolated from cerebral cortex tissue were purchased from Cell-systems (ACBRI 498), where they were tested for bacterial, fungal and mycoplasma sterility by an independent laboratory. The pericyte cell line has been verified by immunofluorescence assays in which >98% were positive for desmin at passage (P)3, PDGFRB at P3 and P10, NG2 at P3 and P10, CD13 at P3 and P10, and α-SMA at P10, while <2% were positive for CD31, MAP2, neurofilament neuronal marker s100A4, GFAP, GS astrocyte marker, CD11b and Iba1 for pericyte characterization. The cells were grown in complete-classic medium supplemented with 10% serum, 5 ml CultureBoost and (2 ng/ml) Bac-Off at 37°C and 5% CO_2_, coated with the attachment factor derived from heat-inactivated charcoal-stripped fetal bovine serum in PBS-based HEPES-buffered gelatine vehicle (Cell-systems). Cells from passage 3-6 were used for experiments.

### Cell transfection

Cells were seeded in culture plates coated with attachment factor at 40-80% confluency as specified for each experiment and left to adhere overnight (O/N). Lipid siRNA complexes were generated with lipofectamine RNAimax reagent (Life Technologies), and siRNA transfection was conducted according to the manufacturer’s protocol. Briefly, cells were washed with PBS and the culture medium was replaced with serum-reduced Opti-MEM medium (Thermo Fisher Scientific). The siRNA complexes and lipofectamine RNAimax were diluted in Opti-MEM before being added in 1:1 ratio and left to incubate at room temperature (RT) for 5 min. Concentrations of diluted siRNA-lipid complexes were scaled according to the manufacturer’s protocol and added to the cells with the final concentrations ranging from 5 to 25 pmol siRNA and 1.5 to 7.5 μl lipofectamine (depending on the number of cells seeded) in 24-well, 12-well or six-well plates. After 6 h at 37°C in the incubator, the medium was replaced with normal complete-classic medium supplemented with 10% serum, 5 ml CultureBoost and Bac-Off (2 ng/ml). The following siRNAs were used: RGS5 silencer siRNA (Ambion, 4392420), HIF-1α silencer siRNA (Ambion, am51331) and silencer negative control #1 siRNA (Ambion, 4390843). Additionally, we used non-siRNA-treated cells as WT control group when indicated.

### Live imaging

#### Chemokinesis

Cells were seeded in a 24-well plate coated with attachment factor at 30% confluency and incubated O/N at 37°C and 5% CO_2_. Cells were transfected with RGS5 siRNA or control siRNA and left to incubate for 24 h before the start of the experiment. First, the cells were pre-incubated for 3 h under oxygen deprivation (0.5-0.7% O_2_) in a humidified, gas-tight hypoxia chamber (Electrotek) with a gas composition of 85% N_2,_ 10% H_2_ and 5% CO_2_. The growth medium was replaced with serum-free Dulbecco's modified Eagle medium (DMEM/F12) supplemented with 15 mM HEPES (Thermo Fischer Scientific) and deoxygenated by pre-bubbling the medium for 15 min in N_2_ gas, generating an oxygen concentration of 0.5-0.7% in the medium when added to the cells. Throughout experiments, Electrotek anaerobic indicator solution was used (containing, 2% w/v C_6_H_12_O_6_, 9% w/v NaHCO_3_ and 1% w/v Methylene Blue solution in water) to monitor O_2_ levels below 1% (Electrotek-scientific). After 3 h, cells were stimulated with 1 µM S1P conjugated to human serum albumin as a carrier protein (Avanti Polar Lipids) or 5 ng/ml PDGFBB (R&D Systems) (Gunaje et al., 2011; Liu et al., 2018; Yu et al., 2000).

Next, cells were directly transferred to the live-imaging microscopy platform cell discoverer CD7 (Zeiss microscopy) with a humidified and hypoxic atmosphere at 37°C in 5% CO_2_ and 0.1% O_2_. Cells were imaged, tracked and averaged from four pre-selected non-overlapping positions per well with 30 min frame intervals for a duration of 24 h using phase contrast and a 5× objective. Cells from three independent experiments were analysed.

#### Image analysis

Cellular trajectories were created by manually tracking cells using the MTrackJ plug-in in ImageJ (National Institutes of Health). The *x*, *y* coordinates were used for further analysis. For quantification of MSD and speed assessment, an open-source computer program (DiPer) and open program source codes were used (Gorelik and Gautreau, 2014).

MSDs were computed by the software according to Eqn 1 using overlapping time intervals. Here, MSD (*n*) is the MSD for a specified cell for step size *n* and *N* is the total number of cell displacements per trajectory. Here, Δt refers to the time interval between adjacent points along the migration trajectory. Population averages were then generated for each time interval *n*Δt where MSDs were averaged over all tracked cells and plotted against the time interval.
(1)

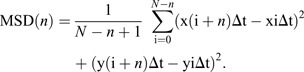
The instantaneous speed was calculated by the software for each cell that was tracked (ν=d/t) for each time frame. Additionally, the AUC was calculated from the instantaneous speed and plotted as bar graphs.

### Chemotaxis

For hypoxic imaging, pericytes (10,000 cells) were seeded on fibronectin (10 μg/ml)-coated chemotaxis µ-slides (Ibidi) and left to adhere O/N. Prior to live imaging, the chemotaxis µ-slides were transferred to the hypoxic chamber, and the reservoirs were filled with deoxygenated (0.5-0.7% O_2_) serum-free DMEM/F12 supplemented with 15 nM HEPES. Chemo-attractants PDGFBB (50 ng/ml) or S1P (1 μM) were applied to the left reservoir after 1 h of hypoxic pre-incubation. The µ-slides were then imaged using the CD7 microscope with a humidified and hypoxic atmosphere at 37°C in 5% CO_2_ and 0.1% O_2_. The chemotactic live imaging acquisition was then conducted during 1-23 h of hypoxia. For normoxic imaging, the same experimental set-up was used, but the live imaging was performed using a Nikon eclipse Ti microscope with a humidified chamber from Okolab at 37°C in 5% CO_2_ with atmospheric oxygen concentrations. Time frame intervals were set to 15 min for the duration of 22 h using a 20× objective with 0.5× magnification for hypoxic imaging, and a 10× objective was used for the normoxic microscope. Subsequently, an equal number of cells from the experimental groups siRGS5 or siCTRL pericytes treated with either PDGFBB or S1P were analysed from three independent experiments. The cells from each experiment were tracked using ImageJ plugin MTrackJ throughout the time period. The *x* and *y* coordinates from the cell tracks were exported to txt format and directly imported, statistically analysed, and computed using the chemotaxis and migration tool 1.01 (Ibidi integrated BioDiagnostics) in ImageJ. The cell trajectories were all extrapolated to *x*, *y*=0 at the starting point of time 0 h.

### Scratch assay

Pericytes were seeded at 60% confluency (25,000 cells/well) on 24-well plates coated with attachment factor and left in the culture O/N. Scratches were introduced 24 h post-transfection at∼100% confluency using a 200 μl pipette tip, and the wells were washed with PBS 3× to avoid cells re-adhering. The scratch was imaged immediately afterwards at time 0 h. Subsequently, cells were transferred to the hypoxic chamber, and deoxygenated (0.5-0.7% O_2_) serum-free DMEM/F12 medium supplemented with 15 nM HEPES was added to the wells. Next, cells were deprived of O_2_ and, after 1 h, treated with either 1 μM S1P or 5 ng/ml PDGFBB. The scratches were imaged again after 8 h of hypoxic insult using 2× objective phase contrast on an Olympus CKX41 microscope. The migration was analysed with ImageJ to determine the rates of cell migration into the scratch wound-healing area by measuring the percentage of wound-healing closure between 0 and 8 h.

### EdU proliferation (cell cycle analysis)

Proliferation evaluation was conducted using Click-iT Plus EdU Flow Cytometry Assay (Invitrogen), according to the manufacturer’s protocol. Briefly, 200,000 cells were seeded in six-well plates coated with attachment factor. Next, 24 h post-siRNA transfection, the cells were deprived of O_2_ (0.5-0.7% O_2_) for 1 h before being stimulated with 1 μM S1P or 5 ng/ml PDGFBB and left for 8 h. During the last hour, 10 μM of EdU was added to the culture medium before the cells were washed 1× with PBS and dislodged using a cell scraper. Next, cells were washed with 1 ml of PBS with bovine serum albumin (BSA) followed by another wash with PBS. Cells were then resuspended in 1 ml PBS with LIVE/DEAD Fixable Dead Cell Stain kit (1:1000, Thermo Fisher Scientific) and incubated on ice for 30 min before washing once with 1 ml of PBS. The cells were then washed with 1 ml of 1% BSA in PBS before addition of 100 μl of 4% paraformaldehyde (PFA) and incubation at RT for 15 min. Cells were then washed with 100 µl of 1% BSA in PBS followed by resuspension in 100 μl of 1× Click-iT permeabilization and wash reagent and incubated for another 15 min at RT. Then, cells were incubated with NG2 antibody (Thermo Fisher Scientific) at 1 μg/ml for 15 min at 4°C, washed with 100 μl of 1% BSA in PBS and resuspended in 33 μl of 1× Click-iT permeabilization and wash reagent. Thereafter, 166 μl of Click-iT Plus reaction cocktail was added to the cells for 30 min at RT before the cells were washed 2× with 100 μl of Click-iT permeabilization and wash reagent. Next, cells were resuspended in 0.5% fetal bovine serum in PBS, 4′,6-diamidino-2-phenylindole (DAPI; 1:10,000, Thermo Fisher Scientific) was added to stain the DNA content, and cells were incubated for 5 min at RT before analysis using a BD LSRII flow cytometer (BD Biosciences). Flow cytometry data were analysed using FlowJo software (FLOWJO LLC, version 10.7.2).

### Western blotting

For western blotting, pericytes were seeded in 24-well plates (30,000 cells/well). The cells were lysed directly in the well using 100 μl of 1× Laemmli buffer (Bio-Rad) supplemented with 0.1 M DTT. The lysates were denatured at 95°C for 5 min and run on 15-well 4-15% SDS-PAGE gels (Bio-Rad) before being blotted onto Turbo-transfer-packs (Bio-Rad). The post-transfer nitrocellulose membranes were then blocked for 1 h in 5% milk in Tris-buffered saline with 0.1% Tween-20 (TBST). Next, the membranes were incubated with the primary antibodies (Table S1) in 5% BSA in TBST O/N. The membranes were subsequently washed 3× with TBST before goat-anti-rabbit-HRP secondary antibody was added (1:5000, Dako) in 5% milk in TBST and incubated at RT for 1 h. Membranes were then washed 3× with TBST followed by protein detection using HRP substrates Clarity or Clarity Max (Bio-Rad) to measure the chemiluminescence on a ChemiDoc (Bio-Rad).

For evaluating signalling mechanisms of PDGFRβ and intracellular phosphorylation of MAPK targets, siRGS5 or siCTRL pericytes were exposed to 8 h of hypoxia in deoxygenated (0.5-0.7% O_2_) serum-free DMEM/F12 medium supplemented with 15 nM HEPES before being stimulated with either S1P (1 μM) or PDGFBB (5 ng/ml) for 5 min, directly lysed and analysed by western blotting. For additional information on antibody validation, see Table S3.

### Real-time qPCR

Pericytes were seeded in 24-well plates (30,000 cells/well) and total RNA was extracted using an RNeasy Mini kit (Qiagen) according to the manufacturer’s protocol. Nanodrop 2000c (Thermo Fisher Scientific) was used to determine the quantity and quality of the RNA with 260/280 and 260/230 ratios. The RNA was reverse transcribed using a Maxima First Strand cDNA Synthesis Kit (Thermo Fisher Scientific), total volume (20 μl). For the qPCR, 10 μl reactions were run with 2 ng of cDNA, 1× SsoAdvanced Universal SYBR Green Supermix and 250 nM of forward and reverse primers.For a list of the primers used in this study, see Table S2.

### Statistical analysis

Statistical analysis was performed using GraphPad Prism software version 8.2.1 or chemotaxis and migration tool 1.01 (Ibidi integrated BioDiagnostics). All statistical tests were performed on independent experiments from the same pericyte cell line indicated as the sample size in the figure legends. The sample size (*n*), statistical test and *P*-values are indicated in the figures or figure legends. The significance (*P*<0.05) was calculated using one-way ANOVA with Tukey's multiple comparisons, multiple *t*-tests or unpaired Student's *t*-test.

## Supplementary Material

10.1242/biolopen.059371_sup1 Supplementary information
