# Supplementary material for: RGS5: a novel role as a hypoxia-responsive protein that suppresses chemokinetic and chemotactic migration in brain pericytes
Source: Biol Open. 2022 Oct 17;11(10):bio059371. doi: 10.1242/bio.059371 (PMC9596146; doi:10.1242/bio.059371)
Supplement: Supplementary information [file biolopen-11-059371-s1.pdf]

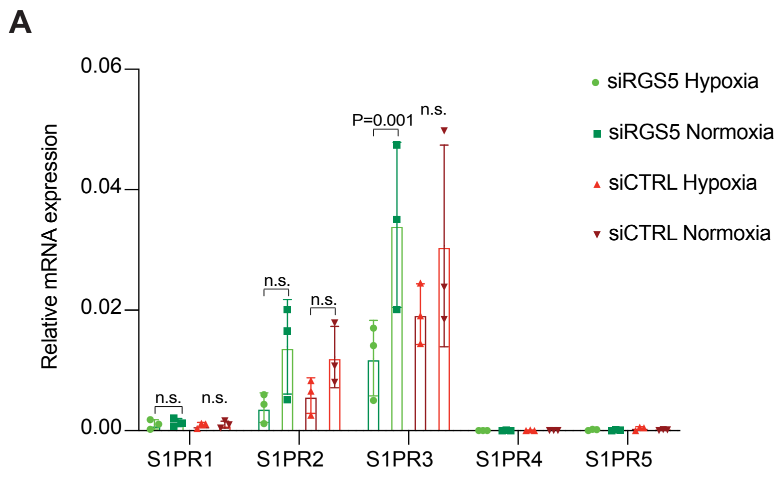

Fig. S1. A Brain pericytes mainly express S1PR2 and S1PR3. A Relative mRNA quantification of S1PR1, S1PR2, S1PR3, S1PR4 and S1PR5 was normalized to β-2 microglobulin (β2M) in either normoxic conditions or after 24 h of hypoxia. Data is presented as mean ± S.D. Statistical analysis was performed using two-way ANOVA with Tukey´s multiple comparisons, n = 3.

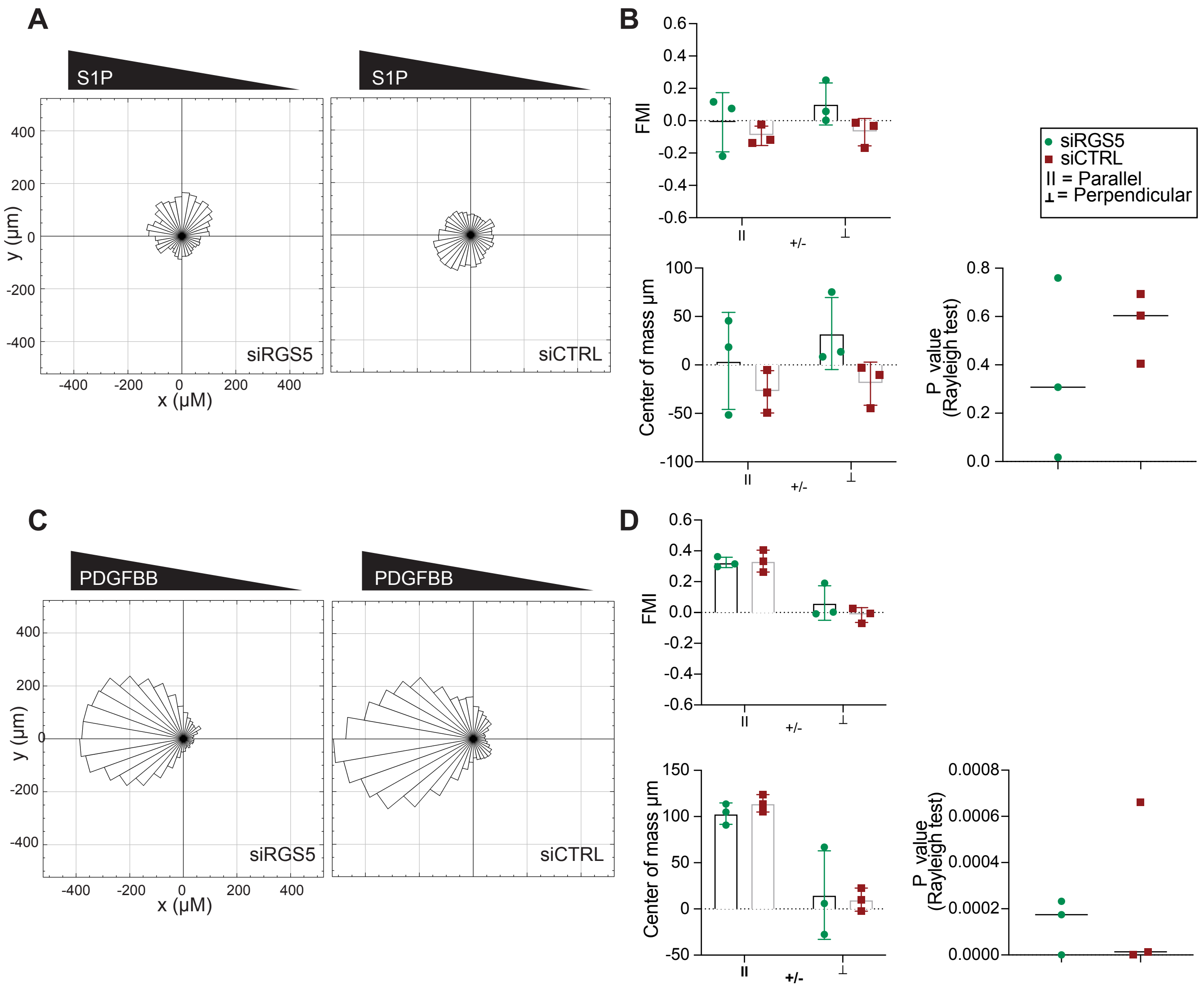

Fig. S2. PDGFBB but not S1P induce chemotaxis in pericytes under normoxia. A Rose plot of S1P (1 µM) treated pericytes transfected with either siRGS5 or siCTRL siRNA under normoxic conditions. The rose plot illustrates 57 siRGS5 treated cells and 56 siCTRL treated cells n=3. B The FMI, centre of mass and circular distribution of cell trajectories were calculated to assess the chemotactic migration in response to a stable linear gradient with the highest concentration of S1P being 1 µM. C Rose plot of PDGFBB (5ng/ml) treated pericytes transfected with either siRGS5 or siCTRL siRNA under normoxic conditions. The rose plot illustrates 83 siRGS5 treated cells and 103 siCTRL treated cells n=3. D The FMI, centre of mass and circular distribution of cell trajectories were calculated to assess the chemotactic migration in response to a stable linear gradient with the highest concentration of PDGFBB being 5 ng/ml. The data is presented as mean ± S.D. and Rayleigh test was computed for the evaluation of circular distribution uniformity of cell trajectory endpoints.

Biology Open • Supplementary information

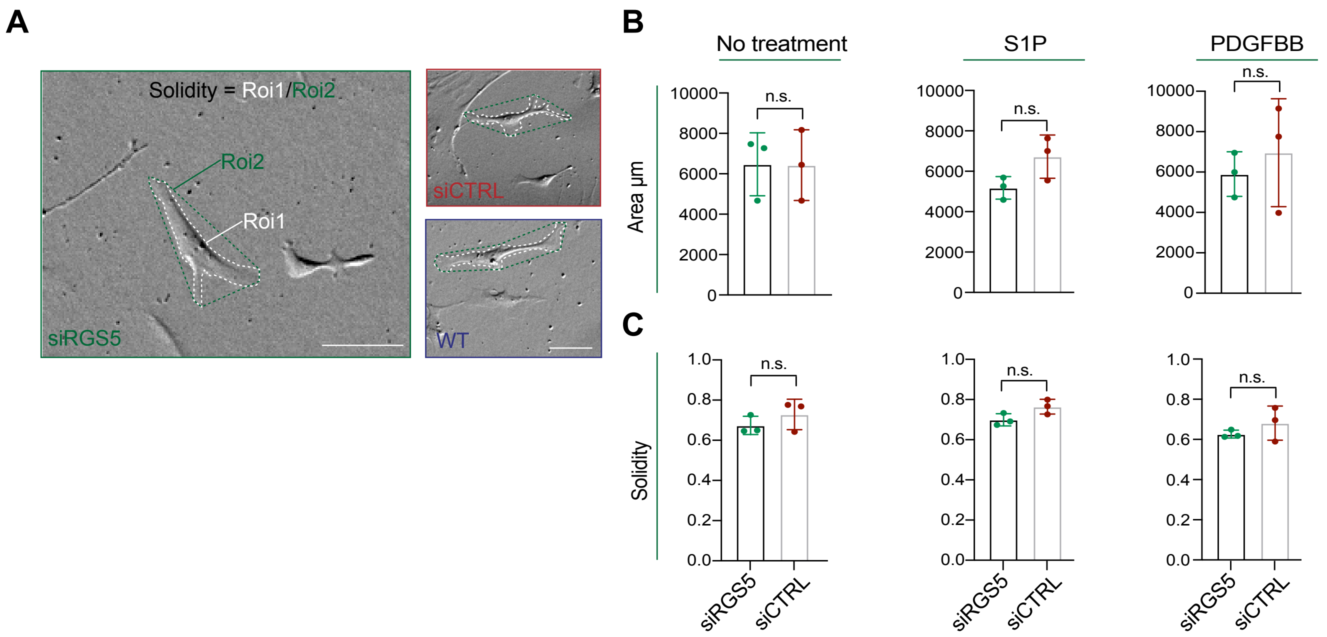

Fig. S3. RGS5 expression does not change the morphology of pericytes under hypoxia. A Illustration of solidity measurement as the area of the cellular border divided by the area including its convex hull after 24 h of hypoxia. B The cell size area was measured by outlining cellular borders of non-treated, S1P (1 µM) and PDGFBB (5ng/ml) treated experimental groups. C Solidity quantification of non-treated, S1P (1 µM) and PDGFBB (5ng/ml) treated experimental groups. The data is presented as mean ± S.D. Statistical analysis was performed using unpaired Student´s t-test. P values are indicated, n = 3. Scale bar 100 µm.

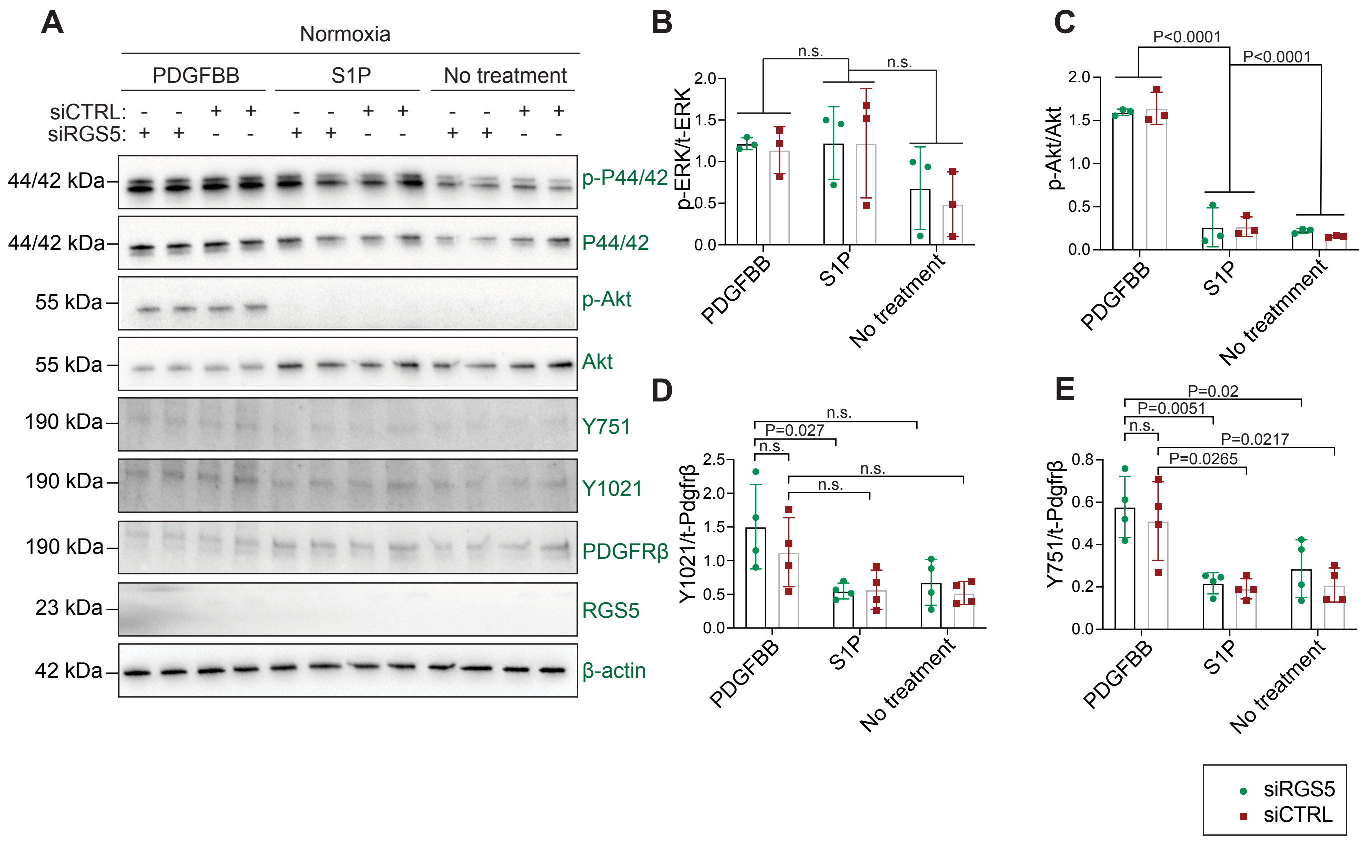

Fig. S4. MAPK-signalling and PDGFRβ-phosphorylation in normoxia. A Western blot of p-ERK/ERK, p-AKT/AKT, p-Y1021, p-Y751 and total PDGFRβ in siRGS5 and siCTRL pericytes treated with S1P (1 µM) or PDGFBB (5 ng/ml) for 5 min or no treatment under normoxic conditions. B-E Quantification of relative phosphorylation of respective target after each treatment. The data is presented as mean ± S.D. Statistical analysis was performed using two-way ANOVA with Tukey's multiple comparisons. P values are indicated, n = 3.

Table S1. List of antibodies used in this study.

Name	Company	Species	Concentration	Cat #
Rgs5	Protein Tech	Rabbit	1:1000	11590-1-AP
p-P44/42	CST	Rabbit	1:1000	9101s
P44/42	CST	Rabbit	1:1000	9102s
p-Akt	CST	Rabbit	1:1000	9271s
Akt	CST	Rabbit	1:1000	9272s
p-Pdgfrβ (Y751)	CST	Rabbit	1:1000	3161s
p-Pdgfrβ (Y1021)	CST	Rabbit	1:1000	2227s
Pdgfrβ	CST	Rabbit	1:1000	4564s
Hif-1α	CST	Rabbit	1:1000	36169s
NG2	Thermo Fischer	Mouse	1 µg/ml	MA5-28549

Table S2. List of primers used in this study

Name	Forward	Reverse	Company
RGS5	GGTCTTGGCTGGTTTCTCTG	GTGCAAAGGACTTGCAGCTT	Eurofins
Hif-1α	AGGGCAGGATACACANGATTTG	TTCCCGACTAGGCCCANTC	TAGC Cph
β2M	CATTCCTGAAGCTGACAGCATTC	TGCTGGATGACGTGAGTAAACC	TAGC Cph
S1PR1	TCTGCGGGAAGGGAGTATGT	CGATGGCGAGGAGACTGAA	Eurofins
S1PR2	CGTTGGCATCAAAGATGGACA	CGATGGCGAGGAGACTGAA	Eurofins
S1PR3	TCTACCATCCTGCCCCTCTAC	ACACGCTCACCACAATCACC	Eurofins
S1PR4	CAACCCCATCATCTACTCCTTCC	AAAGCTGTCCCTTGGCCTC	Eurofins
S1PR5	TGTATTTATGCAGCGACACCCC	CTTCCCTGACCACCATCACC	Eurofins

Table S3. Additional information about antibody validation

Name	Company	Species	Conc	Cat #	Citation/Validation
Rgs5	Protein Tech	Rabbit	1:1000	11590-1-AP	(Quitterer et al., 2019)/We validated the RGS5 antibody using siRNA silencing of the protein by western blotting
p-P44/42	CST	Rabbit	1:1000	9101s	(Arslanbaeva et al., 2022)/ The antibody has been validated on mouse fibroblasts using e.g., PDGFBB stimuli by western blotting
P44/42	CST	Rabbit	1:1000	9102s	(Liu et al., 2022)/ Antibody has been validated by siRNA transfection in HeLa cells by western blotting
p-Akt	CST	Rabbit	1:1000	9271s	(Scognamiglio et al., 2022)/ Antibody has been validated by PDGFBB treatment on NIH/3T3 cells by western blotting
Akt	CST	Rabbit	1:1000	9272s	(Scognamiglio et al., 2022)/ Antibody has been validated using siRNA in CHO cells by western blotting
p-Pdgfrβ (Y751)	CST	Rabbit	1:1000	3161s	(Kubota et al., 2022)/ Antibody has been validated after PDGFBB stimuli in various cell lines (L6/HepG2/NIH/3T3) by western blotting
p-Pdgfrβ (Y1021)	CST	Rabbit	1:1000	2227s	(Dhahri et al., 2016)/ Antibody has been validated after PDGFBB treatment in NIH/3T3 cells by western blotting
Pdgfrβ	CST	Rabbit	1:1000	4564s	(Zhang et al., 2021)/ Antibody has been validated in hSkMC, C2C12 and NIH3T3 cells
Hif-1α	CST	Rabbit	1:1000	36169s	(Chen et al., 2022)/ Antibody has been validated in HepG2 cells treated withb cobalt chloride
NG2	Thermo Fischer	Mouse	1 µg/ml	MA5-28549	Antibody has been validated in SK-MEL-30 and SP2 cells using flow cytometry

Biology Open • Supplementary information
